# 2019 EULAR/ACR classification criteria for SLE score predicts future lupus hospital admission and costs

**DOI:** 10.1177/09612033241310071

**Published:** 2024-12-19

**Authors:** Saurav Suman, Hammad Ali, Connor R Buechler, Heidi C Rogers, W Neal Roberts

**Affiliations:** 1 Division of Rheumatology, 8082 Penn State University, Hershey, PA, USA; 2 Department of Dermatology, 14640 University of Pennsylvania, Philadelphia, PA, USA; 3 Department of Internal Medicine and Dermatology, 12269 University of Minnesota, Minneapolis, MN, USA; 4 Department of Rheumatology, Norton Healthcare, Lousiville, KY, USA; 5 Division of Rheumatology, 12252 University of Kentucky, Lexington, KY, USA

**Keywords:** Lupus erythematosus, systemic, hospitalization cost, hospital admission, cost of lupus care, 2019 EULAR/ACR classification criteria for SLE

## Abstract

**Objective:**

To test the ability of the 2019 EULAR/ACR Classification Criteria for SLE score to predict lupus related hospitalization and overall cost of hospitalization.

**Methods:**

217 University of Kentucky patient records that met our preliminary inclusion criteria, 44 patients were selected by a random number generator algorithm for a thorough chart review to collect data needed for calculation of the 2019 EULAR/ACR Classification Criteria for SLE score. Total hospitalization cost was calculated by using hospital adjusted expenses per inpatient day data, which estimates the expense incurred by the hospital to provide services and thus removes the variability of charges and reimbursements introduced by insurance type.

**Results:**

Patients with a score of 19 or more had increased risk of hospitalization in at least the 6 months after initial outpatient visit as compared to their counterparts with scores less than 19 [*p* = .069]. The odds of being hospitalized for lupus among those with initial score ≥19 was 5.71 times higher than for those with score <19. Patients who scored 19 or less at initial visit had a mean hospitalization cost of $14,499, whereas those scored >19 had mean hospitalization cost of $28,725.

**Conclusion:**

This study adds to the growing evidence that 2019 EULAR/ACR Classification Criteria score for SLE can be used as a surrogate marker to assess disease severity. The weighted 2019 EULAR/ACR Classification Criteria for SLE score offers a promising tool beyond its primary objective to find true lupus cases for research and clinical trials.

There are no specific validated tools to predict the risk of hospitalization in patients with systemic lupus erythematosus (SLE). Several investigations have suggested the “off label” utility of the quantitative score of the 2019 EULAR/ACR Classification Criteria for SLE to capture prognostic information and clinical outcome. Using standard methods such as receiver operating characteristic curves, score cutoffs between 20 and 25 have been shown to predict a variety of clinical outcomes.^1–3^ We tested the ability of the 2019 EULAR/ACR Classification Criteria for SLE score to predict the risk and potential cost of hospitalizations for patients with SLE.

This study was a retrospective cohort study, analyzing patient records to assess the predictive value of the 2019 EULAR/ACR Classification Criteria for SLE on hospitalization risk and costs. Of the 217 University of Kentucky patient records that met our preliminary inclusion criteria. Inclusion criteria included a confirmed diagnosis of SLE according to the 2019 EULAR/ACR Classification Criteria and having at least one outpatient rheumatology visit documented in the University of Kentucky medical records. Only the charts having the needed data were included, to ensure a manageable and detailed analysis. Due to resource constraints and the need for a thorough manual chart review, a representative subset of 44 patients was selected, by a random number generator algorithm. This subset size was chosen to balance the depth of data collection with feasibility of the study. Thorough chart review to collect data needed for calculation of the 2019 EULAR/ACR Classification Criteria for SLE score, information regarding SLE-related hospitalizations, and time interval (months) between first outpatient rheumatology visit and SLE related hospitalization was done. If a patient was not hospitalized, time between the first rheumatology visit and the last outpatient rheumatology follow up was recorded. Reasons for admission were characterized as either directly related to SLE or incidental. Total days spent in the hospital were calculated using the days of admission and discharge. Finally, total hospitalization cost was calculated by using hospital adjusted expenses per inpatient day data, which estimates the expense incurred by the hospital to provide services and thus removes the variability of charges and reimbursement introduced by insurance type.^5^ We chose a score cutoff of 19 as the value that gave the greatest clinical discrimination between patients hospitalized for SLE and those who were not.

Patients with a score of 19 or more had increased risk of hospitalization in at least the 6 months after initial outpatient visit as compared to their counterparts with scores less than 19 [Figure 1; *p* = .069]. The odds of being hospitalized for lupus among those with initial score ≥19 was 5.71 times higher than for those with score <19. One hundred percent of patients with score ≥19 at index rheumatology visit had been admitted for complications related to lupus by 5.5 years after that visit, which was true of less than half of those with initial score <19.

**Figure 1.**
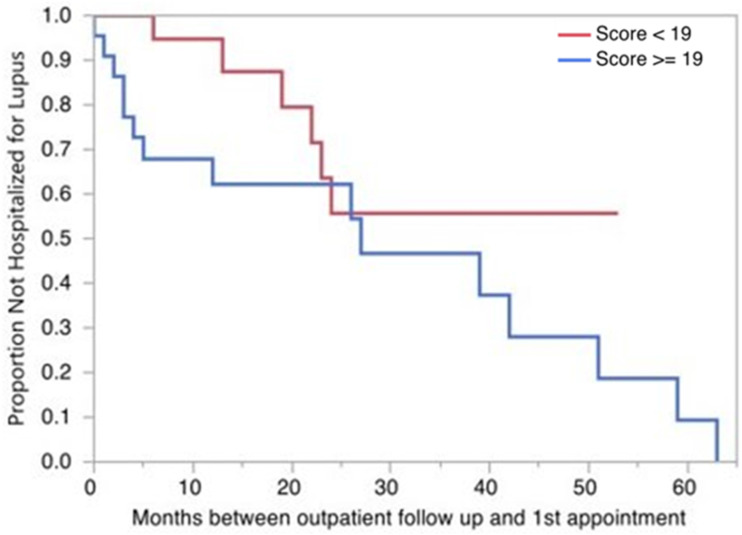
Risk of hospitalization by 2019 EULAR/ACR SLE Classification Criteria. Kaplan- Meier Survival Analysis using the Wilcoxon test is shown, comparing the risk of hospitalization between those with 2019 EULAR/ACR SLE Classification Criteria score of less than 19 (red) and more/equal to 19 (blue) at initial outpatient rheumatology visit.

Among patients hospitalized, 62% were hospitalized within 2 years of their index rheumatology visit. Patients hospitalized due to complications related to lupus had a mean hospital cost of $20,426 in 2020. Those hospitalized for causes unrelated to lupus incurred a mean hospital cost of $17,060.

Patients who scored 19 or less at initial visit had a mean hospitalization cost of $14,499, whereas those scored >19 had mean hospitalization cost of $28,725. Patients with lupus score >19 had almost twice the cost as patients with lupus score 19 or less in the 2 years following their index rheumatology visit.

This study adds to the growing evidence that 2019 EULAR/ACR Classification Criteria score for SLE can be used as a surrogate marker to assess disease severity. Our data is supported by the work of other groups who have found that “criteria counting” with the 2019 EULAR/ACR Classification Criteria for SLE is granular enough to make the criteria themselves prognostic. Most notably, Garcia and colleagues showed that lupus score 20 or more indicated “ominosity” or severity, predicting that these patients were at higher risk for more active disease through the first 5 years following diagnosis.^4^ In our study, we found that a score of ≥19 was associated with increased risk of hospitalization and cost of care. This early prognostic influence waned with time and was not sustained enough to produce a statistical difference beyond 2 years, consistent with the volatile and dynamic nature of SLE.

Our study is the first to our knowledge to quantitatively predict future hospitalization risk and cost of in-hospital care using 2019 EULAR/ACR Classification Criteria for SLE. Our limitations include our sample size and the homogeneity of our hospital expense data, which does not include the cost of specific medications or procedures.

The weighted 2019 EULAR/ACR Classification Criteria for SLE score offers a promising tool beyond its primary objective to find true lupus cases for research and clinical trials. It appears that it can be used to identify patients at elevated risk for organ-threatening disease^4^ and now also those at increased risk of hospitalization and higher cost of care as well. It may soon be possible for electronic medical record applications to gather and collate classification scores to both identify and risk stratify patients with a lupus ICD-10, potentially marking them for a secondary prevention intervention, for example assignment to a nurse navigator and low-cost help to overcome access barriers in both urban and more rural areas. Arguments in favour of such a decision support programming in the Electronic Medical Record will be stronger when grounded in valid predictors of clinical outcomes and hospitalization expense.
